# The relationship between positive exercise experiences and mobile phone addiction tendencies in older adults: a cross-lagged study

**DOI:** 10.3389/fpubh.2025.1710048

**Published:** 2025-11-14

**Authors:** Bin Chen, Wenying Huang, Chang Hu

**Affiliations:** Physical Education College, Jiangxi Normal University, Nanchang, China

**Keywords:** older adults, positive exercise experience, mobile phone addiction, cross-lagged panel model, tracking study

## Abstract

**Background:**

While the literature on mobile phone addiction (MPA) is extensive, it has overwhelmingly focused on younger populations, leaving its manifestation and impact among older adults as a critical research gap. Positive exercise experience (PEE) is a potential protective factor, yet the dynamic, reciprocal relationship between PEE and MPA in later life is poorly understood. This study addresses this gap. This study aimed to (a) examine the stability of PEE and MPA across three time points, (b) explore potential gender differences, and (c) test the reciprocal predictive relationships between PEE and MPA using a cross-lagged panel model.

**Methods:**

A three-wave longitudinal survey (9 months, April 2024–January 2025) was conducted among 828 older adults recruited from senior universities in four provinces of China. Participants completed the Subjective Exercise Experience Scale (PEE dimension) and the Mobile Phone Addiction Tendency Scale. Repeated-measures ANOVA was employed to test temporal and gender effects. Cross-lagged panel analyses were estimated in Mplus 8.0 to assess reciprocal prediction between PEE and MPA.

**Results:**

PEE remained stable across time with no gender differences, whereas MPA showed a significant increase over the three waves but no gender effect. PEE and MPA were significantly negatively correlated at all time points (*r* = −0.513 to −0.136, all *p* < 0.01). Cross-lagged analysis revealed a consistent protective effect: PEE at T₁ predicted lower MPA at T₂ (*β* = −0.110, *p* < 0.01), and PEE at T₂ predicted lower MPA at T₃ (*β* = −0.168, *p* < 0.001). In contrast, MPA at T₁ predicted lower PEE at T₂ (*β* = −0.232, *p* < 0.001), but MPA at T₂ did not predict PEE at T₃.

**Conclusion:**

Findings indicate that PEE is relatively stable in older adults, while MPA increases over time. PEE consistently emerges as a protective factor against future MPA, whereas the inhibitory role of MPA on PEE appears weaker and less stable. These results highlight the importance of promoting enjoyable exercise experiences to mitigate technology-related risks and support healthier aging trajectories.

## 1 Introduction

The pervasive adoption of mobile technologies has precipitated a paradigm shift in the manner in which individuals from diverse age demographics engage with society, communicate with others, and regulate their daily routines (1, 2). Mobile phones, in particular, have become ubiquitous social and personal accessories, providing not only communication tools but also entertainment, information access, and social connection (3, 4). Global surveys indicate that smartphone ownership and daily usage rates among adults are remarkably high, with usage patterns steadily increasing among older populations as well (5). While the benefits of mobile technology are indisputable, this digital expansion has simultaneously given rise to concerns surrounding excessive use (6), often framed as problematic mobile phone use or mobile phone addiction (MPA) (7, 8). Scholars argue that this behavioral pattern, characterized by loss of control, compulsive checking, withdrawal symptoms, and interference with everyday functioning, mirrors other forms of behavioral addictions and deserves serious empirical attention (9, 10).

Early research into mobile phone addiction predominantly focused on adolescents and young adults, groups assumed to be most vulnerable due to their developmental stage, peer influences, and higher receptivity to emerging technologies (11–13). Findings consistently revealed associations between excessive smartphone use and poorer sleep quality, increased depressive symptoms, diminished academic and occupational functioning, and compromised self-regulation (14–16). However, recent demographic shifts demand an extension of this research lens toward older adults (17, 18). The global aging trend, accompanied by increasing rates of technology adoption among older populations, highlights a pressing need to understand how digital dependence manifests in later life (19, 20). Studies across Europe, North America, and China show that older adults are increasingly engaged with mobile phones, and in some cases display problematic patterns comparable to younger cohorts (21–25). Importantly, the mechanisms driving MPA in older adults may differ fundamentally; for instance, where social anxiety often predicts MPA in youth, factors like loneliness, social isolation, or the need to maintain familial connections are more salient drivers in later life (26–28). These factors collectively contribute to high-risk mobile phone addiction behaviors among older adults.

Theoretical models can help explain why mobile phone addiction may emerge as a significant risk among older adults. One potential explanation can be found in the compensatory internet use theory. This theory suggests that individuals may engage in online activities to compensate for deficits or stressors in their offline lives (29, 30). For retirees or widowed older adults, a mobile phone may serve as a convenient surrogate for social support, intimacy, or stimulation (31). Similarly, the social surrogacy hypothesis suggests that media use can compensate for social disconnection, temporarily satisfying belongingness needs (32, 33). Yet, while digital engagement may yield immediate psychological relief, its long-term consequences can be detrimental, reinforcing cycles of dependency and exacerbating loneliness rather than alleviating it (34, 35). Such patterns may impair psychological wellbeing and interfere with health-promoting practices, such as physical activity and social engagement in real-world contexts (36, 37).

Parallel to concerns about MPA, another line of research underscores the value of physical exercise as a multidimensional contributor to wellbeing in older adulthood (38). Exercise has repeatedly been shown to improve physical health outcomes, reduce risk of chronic disease, enhance cognitive function, and alleviate symptoms of depression and anxiety (39–42). Beyond these objective health benefits, an expanding body of literature emphasizes the subjective affective experiences that accompany exercise—namely, feelings of enjoyment, pleasure, accomplishment, and vitality (43–45). These experiences, often referred to as positive exercise experiences (PEE), are increasingly recognized as crucial determinants of sustained physical activity engagement (46). Unlike studies that focus solely on metrics of frequency, duration, or intensity, this perspective highlights the lived emotional and psychological responses to exercise, which can profoundly influence motivation and long-term adherence (47, 48).

Linkages between PEE and reduced MPA have begun to emerge. Several studies suggest that engaging in meaningful, enjoyable physical activity can function as a protective factor against behavioral addictions, including internet and MPA (49, 50). However, it is important to note that these previous studies primarily focused on younger populations—adolescents and university students—leaving the relationship between PEE and MPA in older adults largely unexplored. This represents a critical research gap that our study addresses. While our study focuses on problematic mobile phone use, it is essential to acknowledge that internet and mobile technology use among older adults is not inherently negative. Evidence suggests that appropriate use of digital technologies can promote social connection, reduce feelings of isolation, facilitate communication with family members, and provide access to health information and services (51). The distinction between beneficial use and problematic addiction is therefore crucial. Our study specifically examines the latter—patterns of use characterized by loss of control, interference with daily functioning, and withdrawal symptoms—which may undermine wellbeing despite the potential benefits of moderate digital engagement. The mechanism is likely multifaceted. According to self-determination theory, positive exercise experiences meet three basic psychological needs: autonomy (the sense of volition and choice), competence (a sense of effectiveness and mastery), and relatedness (feelings of connection with others) (52). When these needs are satisfied, individuals are less likely to seek alternative, technology-mediated avenues for fulfillment (53). Exercise can therefore serve as both a preventive and corrective strategy, helping reduce the emotional vulnerabilities that feed into digital dependence.

The underlying mechanisms linking physical activity to a reduced risk for behavioral addictions are multifaceted. From a neurobiological perspective, exercise is understood to modulate dopamine-related reward pathways in the brain, functioning as a natural and healthy source of reward that may compete with the instant gratification derived from excessive mobile phone use (54). Furthermore, from a psychological standpoint, regular physical activity has been shown to improve executive functions, including self-regulation and impulse control—cognitive skills that are often compromised in individuals with addictive behaviors (55). This dual impact provides a robust theoretical basis for hypothesizing that positive exercise experiences may serve as a protective factor against the development of mobile phone addiction.

In addition, evidence supporting this claim is accumulating across diverse contexts. International research has demonstrated that regular PEE not only enhances physiological health but also fosters psychological resilience and independence in older populations (56–58). For instance, longitudinal work indicates that older adults who engage in exercise report enhanced self-efficacy, which in turn reduces reliance on quick but potentially harmful sources of gratification, such as mobile phone use (59). Other studies highlight the social dimension: participation in group exercise programs enriches interpersonal networks, mitigates loneliness, and diminishes the compensatory appeal of online interactions (60, 61). In addition, the affective benefits of exercise—such as improvements in mood, emotional regulation, and stress recovery—may counteract the very deficits that often drive individuals into compulsive smartphone use (62).

At the same time, the relationship between PEE and MPA may be bidirectional. While positive exercise experiences can alleviate MPA, MPA may, conversely, undermine the potential for such PEE. Excessive smartphone use can monopolize attention, fragment daily routines, and constrain opportunities for physical activity (63). Empirical studies have found that MPA correlates with sedentary behavior and diminished motivation for exercise, suggesting that screen-based gratifications may crowd out the appeal of embodied activities (64). Further, addiction has been associated with impaired emotional processing, meaning that individuals who are excessively reliant on their mobile phones may lose sensitivity to the subtle rewards of physical activity (65). Thus, MPA may blunt the ability to recognize or savor positive exercise experiences, creating a potentially vicious cycle. This bidirectionality underscores the importance of investigating not only direct pathways from exercise to addiction but also reciprocal influences over time.

Gender represents an additional dimension of complexity. The literature reveals mixed findings on whether men and women differ in exercise-related affect and MPA. Historically, men have been shown to report higher levels of exercise enjoyment, reflecting broader patterns of male dominance in sports and leisure activities (66). However, more recent evidence suggests narrowing gender gaps, particularly in later adulthood, where participation opportunities are increasingly equalized (67). Regarding MPA, some studies indicate higher tendencies among women, potentially linked to differences in social use patterns, while others find no significant gender effect (68, 69). The lack of consensus highlights the need to examine whether gender moderates the relationship between PEE and MPA, particularly in populations of older adults who may differ substantially from younger cohorts in lifestyle and technology usage. Therefore, while not the primary aim, exploratorily examining gender differences is crucial for a nuanced understanding of this dynamic in a population where such data remains scarce.

Despite the evident relevance, most existing investigations into PEE and MPA have been cross-sectional. Cross-sectional designs provide important descriptive associations but are limited in establishing causal or temporal directionality. A significant unanswered question is whether PEE reduces smartphone addiction over time, whether MPA diminishes subsequent exercise experiences, or whether both processes occur in tandem. To address such questions, more rigorous longitudinal methods are required. The cross-lagged panel design offers a powerful framework in this regard. By measuring variables repeatedly over multiple time points, researchers can assess reciprocal influences while accounting for stability and prior levels of each construct. Longitudinal data are therefore essential to disentangle the dynamic interplay between PEE and MPA in older adults.

Building on these theoretical foundations and addressing gaps in prior research, the present study employs a three-wave longitudinal design to explore dynamic associations between PEE and MPA among older adults. Specifically, we tested four hypotheses: (H1) both PEE and MPA would exhibit temporal stability; (H2) gender differences would emerge in these constructs; (H3) PEE at earlier waves would predict lower subsequent MPA; and (H4) MPA at earlier waves would predict lower subsequent PEE. By adopting a cross-lagged approach, this study seeks to clarify the bidirectional nature of these relationships and provide empirical insights into pathways that may inform interventions for healthier aging.

## 2 Materials and methods

### 2.1 Participants and procedure

This study adopted a three-wave longitudinal tracking design over a nine-month period. *A priori* power analysis was conducted using G*Power 3.1 to determine the minimum required sample size for a repeated-measures ANOVA with between-factors. The input parameters were set as follows: effect size *f* = 0.25, *α* error probability = 0.05, power (1–*β*) = 0.95, number of groups = 2, number of measurements = 3, correlation among repeated measures = 0, and nonsphericity correction *ε* = 0.5. The effect size was set at f = 0.25, which, according to Cohen’s (1992) guidelines (70), represents a medium effect size. This value was chosen as a conservative and meaningful threshold for detecting practical significance in behavioral science research when prior longitudinal data are not available to inform a more precise estimate. The analysis indicated a minimum sample size of 142 participants. To ensure sufficient statistical power and account for potential attrition over three measurement points, we recruited a substantially larger sample.

We recruited from senior universities to access active, digitally literate older adults, enable structured three-wave follow-up, and study established smartphone use patterns; however, generalizability to less active or digitally engaged community elders is limited. We set the age threshold at 60 + based on China’s retirement age, WHO-aligned definitions, and prior Chinese research (25). Acknowledging aging heterogeneity, early-60s differ from those in their 70s–80s in function, health, and adoption. Our sample is relatively healthy and active, which should inform interpretation.

A multi-stage convenience sampling strategy was employed. First, four provinces (Jiangxi, Hunan, Guizhou, and Chongqing) were selected based on accessibility and established research collaborations that provided access to the target population. Within each province, administrators at senior universities were contacted, and all institutions that formally agreed to participate were included in the sample. Inclusion criteria were as follows: (a) aged 60 years or older; (b) currently enrolled in a senior university program; (c) able to understand the study procedures and provide informed consent; and (d) possessing basic skills in smartphone use, ability to send and receive text messages or use messaging applications (e.g., WeChat), and capability to independently operate basic smartphone functions such as making calls and accessing applications. This criterion was assessed through a brief verbal screening conducted by senior university staff during the recruitment phase, rather than through formal skills testing. Exclusion criteria included: (a) cognitive or sensory impairments preventing completion of the survey; (b) severe physical or psychological illness interfering with participation; and (c) incomplete data across all three measurement points. During each data collection session, trained research assistants were present in the classroom to distribute materials, provide standardized instructions, answer clarification questions, and collect completed questionnaires. To minimize potential coercive pressure, assistants were instructed to: (1) maintain a non-intrusive presence by positioning themselves at the back or side of the classroom; (2) avoid monitoring individual participants or observing their responses.

To reduce potential confounding by festive or leisure-related behaviors, all assessments were scheduled on weekdays while deliberately avoiding major Chinese public holidays. Time 1 (T₁): April 15–19, 2024. Time 2 (T₂): September 12–16, 2024. Time 3 (T₃): January 6–10, 2025. In each wave, data were collected through paper questionnaires. Testing sessions were organized within the classroom settings of senior universities. Prior to each session, participants were reminded of the voluntary nature of the study, the anonymity of their responses, and their right to discontinue at any point. In order to ensure the maintenance of fidelity and the minimization of potential bias, the team of trained administrators followed a standardized protocol at all sites. This protocol included a script for introducing the study and guidelines for answering participant questions (e.g., “Please circle one number per line”) or resolving non-substantive technical issues without influencing their responses. This process ensured methodological consistency across all three waves and locations. To enable tracking of participants across the three waves while maintaining anonymity, each participant was assigned a unique identification code at T1. These codes were generated based on a combination of non-identifying demographic information (e.g., last four digits of phone number, birth month and day, first letter of mother’s name) that could not reveal personal identity but allowed for reliable matching across waves. Participants recorded their unique code on each questionnaire at T1, T2, and T3. During each data collection session, trained research assistants were present in the classroom to distribute materials, provide standardized instructions, answer clarification questions, and collect completed questionnaires. To minimize potential coercive pressure, assistants were instructed to: (1) maintain a non-intrusive presence by positioning themselves at the back or side of the classroom; (2) avoid monitoring individual participants or observing their responses. After each wave of data collection, a multi-step screening procedure was applied. First, questionnaires with more than 10% missing responses were removed. Second, patterned or invariant responses (e.g., selecting the same option across all items) were flagged and excluded. Third, extreme completion times (i.e., < 2 min for the scales) were treated as invalid entries. Finally, only participants who completed all three waves and could be matched through unique codes were retained for the longitudinal analyses. This procedure ensured that the final dataset was both reliable and representative of the target population.

The first survey (T_1_) collected 1,342 questionnaires, with 1,154 valid responses retained after screening, yielding an effective recovery rate of 85.9%. The second survey (T_2_) collected 1,006 questionnaires using the same screening criteria as T1, retaining 859 valid responses with a recovery rate of 85.4%. The third survey (T_3_) collected 842 questionnaires. Following the same principles as above, 828 valid questionnaires were retained, yielding a questionnaire recovery rate of 98.3%. The 828 older adult participants included in the final longitudinal sample had an overall retention rate of 71.7% over the nine-month study period. Men accounted for 46.1% of the respondents, while women represented 53.9%. In terms of geographical distribution, nearly one-third came from Jiangxi (29.3%), followed by Guizhou (26.1%), Chongqing (23.0%), and Hunan (21.6%). Most participants resided in urban areas (70.3%), with the remainder living in rural regions (29.7%). Regarding educational attainment, 34.2% reported primary schooling or below, 43.0% reached junior high level, and a smaller proportion had completed high school or technical secondary education (12.4%) or held a college degree and above (10.4%). Monthly income also varied: more than one-third earned no more than RMB 2000 (35.7%), 27.7% reported 2001–3,000 yuan, 25.1% fell in the 3,001–4,000 yuan range, and 11.5% exceeded 4,000 yuan (Table 1).

**Table 1:** Comprehensive characteristics of study participants (*N* = 828).

Characteristic	Category	N	%
Gender	Male	382	46.10%
	Female	446	53.90%
Residence	Rural	246	29.70%
	Urban	582	70.30%
Province	Jiangxi	243	29.3
	Guizhou	216	26.1
	Chongqing	190	23
	Hunan	179	21.6
Education	Primary school or below	283	34.20%
	Junior high	356	43.00%
	High school/technical	103	12.40%
	College or above	86	10.40%
Monthly income (RMB)	≤2000	296	35.70%
	2001–3,000	229	27.70%
	3,001–4,000	208	25.10%
	>4,000	95	11.50%

For each completed wave, participants received monetary compensation of 5 RMB (approximately USD 0.70). The small compensation is only for transportation and time compensation, and does not constitute a strong inducement. The research protocol was subjected to a rigorous review process, after which it was approved by the Institutional Review Board of the School of Physical Education at Jiangxi Normal University (IRP-JXNU-PEC-2024019). All procedures complied with the ethical standards of the Declaration of Helsinki. Written informed consent was obtained from all participants before enrollment.

### 2.2 Measures

#### 2.2.1 Positive exercise experience

Positive exercise experience was assessed using the Subjective Exercise Experiences Scale (SEES) originally developed by McAuley and Courneya (71). For the purpose of this study, only the positive wellbeing subscale was adopted, which captures individuals’ pleasant feelings during exercise sessions. We selected only this subscale because: (1) our framework focused specifically on positive affective experiences as a protective factor; (2) previous Chinese studies have successfully used individual SEES subscales (72); and (3) this minimized respondent burden across three time points. The subscale under consideration consists of four items, which are to be rated on a seven-point Likert scale (1 = strongly disagree to 7 = strongly agree). Higher scores on this scale thus denote more favorable exercise-related affect. In the present sample, the internal consistency was found to be excellent, with Cronbach’s *α* coefficients of 0.961 (T₁), 0.914 (T₂), and 0.804 (T₃).

#### 2.2.2 Mobile phone addiction tendency

MPA was measured with the Mobile Phone Addiction Tendency Scale (MPATS) developed by Xiong and colleagues (73). The MPATS was specifically developed and validated with Chinese populations, ensuring linguistic and cultural appropriateness. The instrument consists of 16 items covering four domains: withdrawal symptoms, salience behavior, social soothing, and mood modification. Items are rated on a five-point Likert scale (1 = strongly disagree to 5 = strongly agree), with higher scores reflecting greater addiction tendency. Reliability was satisfactory, with Cronbach’s *α* = 0.880 (T₁), 0.947 (T₂), and 0.862 (T₃).

### 2.3 Data analysis

Data analyses were carried out in two stages. First, all valid responses from the three measurement waves were entered into SPSS 26.0. Descriptive statistics were calculated for each variable across the three time points. To examine potential gender and time effects, a two-way repeated measures ANOVA was performed separately for positive exercise experience (PEE) and mobile phone addiction (MPA), with time (T₁, T₂, T₃) as the within-subjects factor and gender (male, female) as the between-subjects factor. When the sphericity assumption was violated, Greenhouse–Geisser corrections were applied. Significance was set at the conventional level of *p* < 0.05.

Second, prior to modeling longitudinal associations, longitudinal measurement invariance was tested for both the Positive Exercise Experience (PEE) and Mobile Phone Addiction (MPA) scales across the three time points using Mplus 8.0. This involved sequentially assessing configural invariance (same factor structure), weak (metric) invariance (equal factor loadings), and strong (scalar) invariance (equal item intercepts). Model fit was evaluated using chi-square (χ^2^), degrees of freedom (df), Comparative Fit Index (CFI), Tucker-Lewis Index (TLI), Standardized Root Mean Square Residual (SRMR), and Root Mean Square Error of Approximation (RMSEA). Invariance was accepted if changes in fit indices between nested models met established criteria (ΔCFI ≤ 0.015, ΔRMSEA ≤ 0.01). To investigate the longitudinal associations between PEE and MPA, a three-wave cross-lagged panel model (CLPM) was specified and estimated in Mplus 8.0. The model included autoregressive effects, which capture the temporal stability of each construct across subsequent waves, and cross-lagged effects, which assess the predictive influence of one construct on the other over time. Specifically, PEE at an earlier time point was modeled as a predictor of MPA at the following time point, and MPA was modeled as a predictor of PEE in the same way. Standardized regression coefficients (*β*) were reported to assess the strength and direction of these longitudinal associations.

This analytic strategy enabled the study to address four main objectives: (a) the temporal stability of PEE and MPA across three waves, (b) potential gender differences in both constructs, (c) whether PEE negatively predicts subsequent MPA, and (d) whether MPA undermines subsequent PEE.

## 3 Results

### 3.1 Common method bias

The Harman’s single-factor test was applied to examine common method bias in the collected data. The results indicated that, under the unrotated exploratory factor analysis, the variance explained by the first factor was 27.05%. None exceeded the 30% threshold (74). According to the criteria for common method bias, the deviations are within an acceptable range.

### 3.2 Measurement invariance analysis

Before testing the main hypotheses, we conducted a longitudinal measurement invariance test across the three time points for the Positive Exercise Experience and Mobile Phone Addiction scales, assessing configural, weak (metric), and strong (scalar) invariance. As shown in the table below, the results indicated a good fit for the strong invariance model for both scales. The changes in CFI and RMSEA between nested models were minimal (ΔCFI ≤ 0.015, ΔRMSEA ≤ 0.01), well within the established criteria for accepting invariance. This confirms that the factor structure, factor loadings, and item intercepts of both scales remained stable over the nine-month period, supporting the validity of the subsequent longitudinal analyses (Table 2).

**Table 2:** Testing for longitudinal invariance of data from three surveys.

Variable	Model	χ^2^	*df*	CFI	TLI	SRMR	RMSEA	Model comparison	ΔCFI	ΔRMSEA
PEE	M1	54.073	39	0.998	0.997	0.015	0.022			
	M2	59.001	45	0.998	0.997	0.018	0.019	M2-M1	0	−0.003
	M3	63.484	53	0.996	0.995	0.019	0.018	M3-M2	−0.002	−0.01
MPA	M1	489.361	186	0.957	0.950	0.056	0.042			
	M2	516.914	202	0.954	0.949	0.059	0.043	M2-M1	−0.003	0.001
	M3	548.523	218	0.949	0.946	0.061	0.045	M3-M2	−0.005	0.002

M1, Configural Invariance; M2, Weak Invariance; M3, Strong Invariance; PEE, Positive Exercise Experience; MPA, Mobile Phone Addiction.

### 3.3 Stability and gender differences in positive exercise experience and mobile phone addiction among older adults

A two-way repeated measures ANOVA with time and gender as factors was conducted. For positive exercise experience, the main effects of time were not significant, *F*(2, 826) = 0.479, *p* > 0.05. The main effects of gender were also nonsignificant, *F*(1, 826) = 0.282, *p* > 0.05, and the interaction effect of time × gender was nonsignificant, *F*(2, 826) = 1.447, *p* > 0.05.

For mobile phone addiction, results indicated a significant main effect of time, *F*(2, 826) = 25.360, *p* < 0.001. However, the main effect of gender was not significant, *F*(1, 826) = 0.803, *p* > 0.05, and the interaction effect of time × gender was nonsignificant, *F*(2, 826) = 0.945, *p* > 0.05. Means and standard deviations are shown in Table 3.

**Table 3:** Means and standard deviations of positive exercise experience and mobile phone addiction in older adults of both sexes.

Gender	T_1_ PEE	T_2_ PEE	T_3_ PEE	T_1_ MPA	T_2_ MPA	T_3_ MPA
Male	3.946 ± 2.115	3.785 ± 1.903	3.766 ± 1.586	3.185 ± 0.811	3.194 ± 1.080	3.342 ± 0.771
Female	3.851 ± 2.123	3.920 ± 1.956	3.886 ± 1.633	3.206 ± 0.846	3.218 ± 1.108	3.438 ± 0.782

T_1_, T_2_ and T_3_ represent the number of measurements.

### 3.4 Means, standard deviations, and correlations of study variables

Descriptive statistics and correlation analyses were conducted. The results demonstrated that within and across the three time points, the correlations among positive exercise experience and mobile phone addiction were all significant. Specifically, positive exercise experience was negatively correlated with mobile phone addiction across all three waves (T₁, T₂, T₃). These patterns suggest a close link between the two variables. Theoretically, this stable negative association provides preliminary cross-sectional support for our central hypothesis. It suggests that the intrinsic rewards gained from positive exercise experiences and the gratifications derived from mobile phone use may function as competing forces for older adults (see Table 4).

**Table 4:** Mean, standard deviation, and correlation matrix of positive exercise experiences and mobile phone addiction in older adults of both sexes.

Variable	M	SD	T_1_ PEE	T_2_ PEE	T_3_ PEE	T_1_ MPA	T_2_ MPA	T_3_ MPA
T_1_ PEE	3.895	2.186	1					
T_2_ PEE	3.858	1.932	0.499^**^	1				
T_3_ PEE	3.831	1.612	0.138^**^	0.412^**^	1			
T_1_ MPA	3.196	0.830	−0.513^**^	−0.427^**^	−0.207^**^	1		
T_2_ MPA	3.207	1.095	−0.379^**^	−0.291^**^	−0.137^**^	0.581^**^	1	
T_3_ MPA	3.394	0.778	−0.380^**^	−0.307^**^	−0.136^**^	0.535^**^	0.529^**^	1

^**^p < 0.01; M, Mean; SD, standard deviation.

### 3.5 Cross-lagged analyses between positive exercise experience and mobile phone addiction

A three-wave cross-lagged panel model was employed to test reciprocal longitudinal relations between positive exercise experience (PEE) and mobile phone addiction (MPA) among older adults.

Autoregressive effects for both constructs were significant and robust. PEE showed strong temporal stability, with scores at T_1_ significantly predicting T_2_ (*β* = 0.380, *p* < 0.001) and T_2_ predicting T_3_ (*β* = 0.407, *p* < 0.001). Similarly, MPA displayed marked stability over time, with significant paths from T_1_ to T_2_ (*β* = 0.525, *p* < 0.001) and from T_2_ to T_3_ (*β* = 0.481, *p* < 0.001).

Cross-lagged effects revealed asymmetric bidirectional associations. For the first interval, higher PEE at T_1_ predicted lower MPA at T_2_ (*β* = −0.110, *p* < 0.01), and higher MPA at T_1_ predicted diminished PEE at T_2_ (*β* = −0.232, *p* < 0.001). Across the second interval, PEE at T_2_ continued to significantly predict reduced MPA at T3 (*β* = −0.168, *p* < 0.001). However, MPA at T_2_ did not significantly predict PEE at T_3_ (*β* = −0.018, *p* > 0.05).

**Figure 1:**
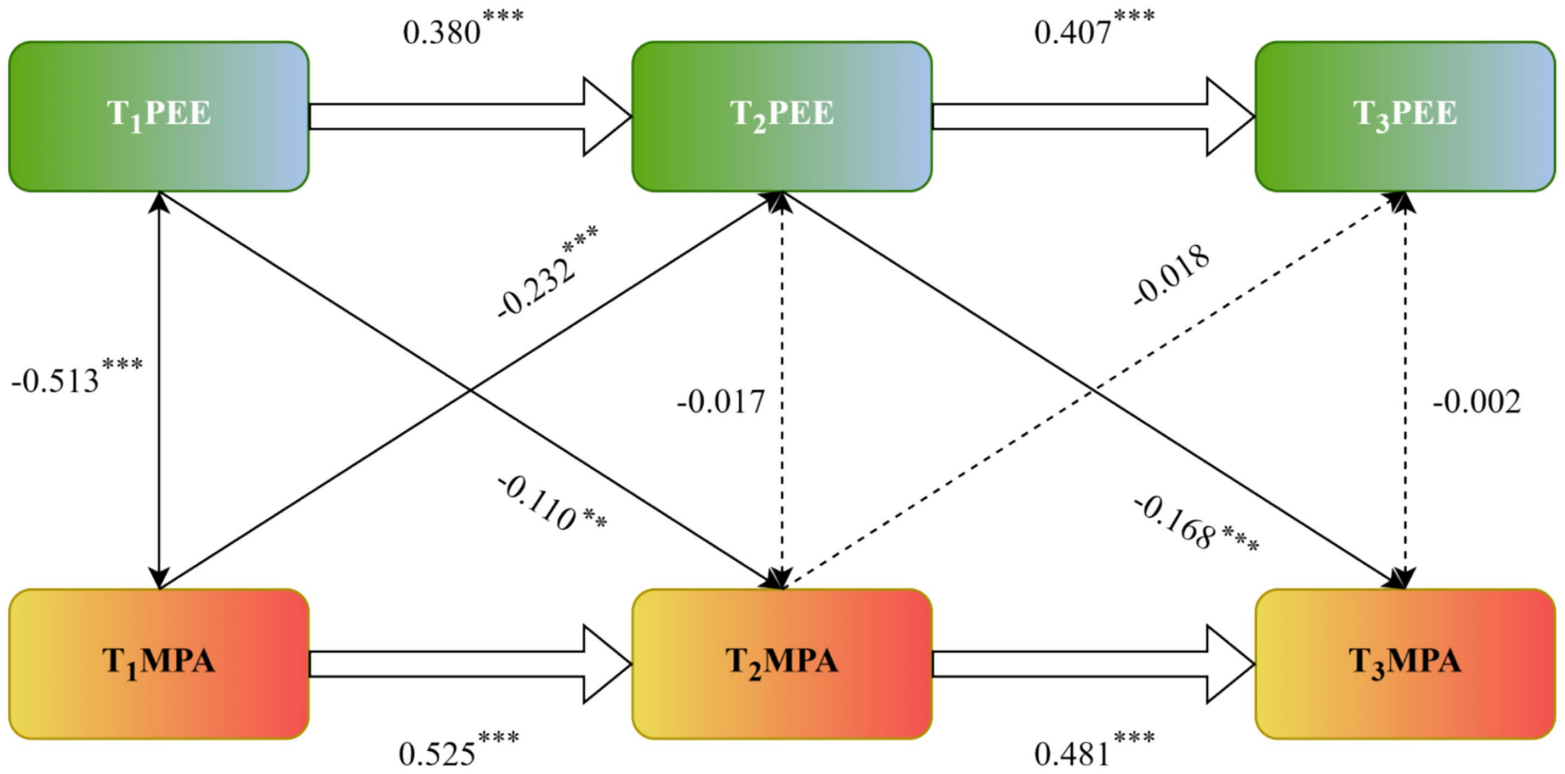
Cross-lagged outcome analysis of positive exercise experiences and mobile phone addiction in older adults. **p < 0.01, ***p < 0.001.

Taken together, these findings indicate that PEE and MPA are negatively interrelated over time, especially in the earlier phase (T_1_ → T_2_), where reciprocal influences were observed. Over subsequent time points, the cross-lagged influence from MPA to PEE weakened and disappeared, while PEE consistently retained its protective effect against later MPA (see Figure 1).

## 4 Discussion

This longitudinal study found that positive exercise experience (PEE) remained stable across 9 months, whereas mobile phone addiction (MPA) significantly increased among older adults. PEE consistently predicted decreases in subsequent MPA, while the effect of MPA on PEE was weaker and less persistent. Gender differences were not observed.

### 4.1 Stability and gender differences

The first objective was to assess the temporal stability of PEE and MPA, as well as potential gender differences. Results indicated that PEE did not vary significantly across time nor between male and female participants. This suggests that, for older adults, the affective component of exercise tends to remain relatively stable, unaffected by short-term contextual fluctuations or gender differences. Such findings align with previous research showing that affective responses to exercise may become more trait-like with age, especially once individuals have established consistent exercise habits (75).

In contrast, MPA showed a significant main effect of time: overall levels tended to increase across the three waves. This result highlights a concerning trend that, rather than diminishing with age, problematic phone use may accumulate over time among older adults. Notably, there was no significant gender effect, indicating that in this population, both men and women are similarly vulnerable to problematic engagement with mobile phones. This finding diverges from studies in younger groups that sometimes report higher addiction rates in women, possibly reflecting age-related convergence in behavioral patterns (76, 77).

### 4.2 Correlations between positive exercise experience and mobile phone addiction

Consistent with expectations, correlations across all three time points demonstrated a significant negative association between PEE and MPA. Older adults who reported higher levels of enjoyment and wellbeing during exercise tended to display lower tendencies toward problematic phone use. This pattern reinforces the notion that positive affective experiences derived from physical activity may provide a protective buffer against compulsive engagement with digital devices.

One explanation lies in the motivational mechanisms proposed by self-determination theory, which emphasizes the fulfillment of autonomy, competence, and relatedness needs (78, 79). When older adults find exercise intrinsically rewarding, the satisfaction of these psychological needs reduces the appeal of alternative spheres—such as smartphone overuse—to compensate for unmet needs (80). Additionally, positive exercise experiences often bring immediate emotional benefits, such as improved mood and reduced stress, making reliance on digital devices for emotional regulation less necessary (81, 82).

The strength of this negative relationship varied over time, with stronger associations observed at the earlier waves and weaker correlations at the final time point. This attenuation may suggest the influence of contextual factors. In China, for instance, the cultural emphasis on family connectivity often intensifies during holidays, potentially increasing mobile phone use for communication (83). Simultaneously, group-based exercise, such as square dancing, is a highly popular and social form of physical activity among older adults (84). The interplay of these cultural norms could shape the relationship between PEE and MPA, and our findings may reflect a context where both digital connection and social exercise are highly valued. The gradual weakening of the correlation could indicate that without ongoing reinforcement, the protective function of PEE might diminish, highlighting the importance of sustained engagement in exercise programs.

### 4.3 Cross-lagged associations between PEE and MPA

The cross-lagged panel analysis revealed reciprocal influences between PEE and MPA, though the strength and direction of effects varied across time. In the first interval, higher PEE at T₁ predicted lower MPA at T₂, while higher MPA at T₁ predicted lower PEE at T₂. This suggests a bidirectional relationship at the early stage of the study: engaging in rewarding exercise experiences helped reduce subsequent addictive tendencies, while elevated phone dependence undermined the ability to derive enjoyment from exercise.

In the second interval, however, only the path from PEE at T₂ to MPA at T₃ remained significant, while the reverse path from MPA to PEE was no longer predictive. This asymmetry implies that the protective effect of positive exercise experiences may persist over time, whereas the negative effect of phone addiction on exercise enjoyment weakens. Several mechanisms could explain this diminishing effect. First, once individuals are already immersed in regular exercise routines, the disruptive influence of smartphone overuse on exercise affect becomes less salient, while the benefits of exercise experiences continue to be reinforced (85). Second, our sample was drawn from senior universities, where participants are typically immersed in structured classes and group activities that provide instructor guidance, peer support, and achievement feedback. Such socially reinforcing contexts may help sustain motivation for offline activities (86). Finally, we posit that once PEE are firmly established and repeatedly reinforced—by satisfying basic psychological needs (autonomy, competence, relatedness) and by receiving social and contextual reinforcement—PEE may become relatively stable and self-reinforcing.

From a theoretical perspective, these results resonate with flow theory (87) and the reward mechanism (88). Exercise that provides positive affect can facilitate immersive engagement, diverting attention away from digital distractions and making phone reliance less compelling. At the same time, the reward response generated during exercise may serve as a healthier source of gratification, replacing the short-lived rewards generated by excessive smartphone use (89, 90). Conversely, the initial inhibitory effect of phone addiction on exercise enjoyment may reflect the compensatory internet use theory, where individuals turn to phones to cope with deficits in offline life, thereby reducing both time and motivation for physical activity (91–93). Over time, however, such compensatory processes may lose strength as daily routines and social contexts shift.

### 4.4 Theoretical implications

Our findings contribute to and refine several theoretical models. Regarding Self-Determination Theory (SDT), the sustained protective effect of PEE on MPA suggests that the fulfillment of basic psychological needs (autonomy, competence, relatedness) through exercise provides a robust and lasting buffer against the allure of digital dependency in later life. This highlights the unique role of intrinsically rewarding activities in promoting long-term self-regulation among older adults.

Furthermore, our results add nuance to the Compensatory Internet Use Theory. While the initial reciprocal relationship supports the model’s premise that individuals use technology to compensate for offline deficits, the disappearance of the path from MPA to PEE over time is noteworthy. This may suggest that for older adults already engaged in a rewarding activity like exercise, a process of adaptation occurs where the negative influence of phone use on exercise enjoyment diminishes. This challenges the assumption that compensatory mechanisms remain stable and points to the potential for positive offline activities to become resilient to the influence of online habits.

### 4.5 Practical implications

The findings carry several implications for promoting healthy aging in the digital era. First, they underscore the potential of positive exercise experiences as a protective factor against problematic phone use in later life. Interventions targeting older adults should therefore extend beyond emphasizing physical benefits to actively cultivating enjoyment, social connection, and psychological satisfaction during exercise programs. Rather than focusing solely on physical outcomes or exercise volume, such interventions should prioritize the cultivation of positive affective experiences through several key principles: creating enjoyable rather than prescriptive exercise environments; incorporating social elements that foster connection and belonging; providing opportunities for autonomy and choice in activity selection; ensuring appropriate difficulty levels that build competence and self-efficacy; and emphasizing immediate emotional benefits (improved mood, vitality, stress reduction) over distant health outcomes.

Although our study did not compare specific exercise modalities, these principles are broadly applicable across various contexts. Group-based activities emphasizing social interaction, mind–body practices integrating movement with mindfulness and relaxation, or recreational activities prioritizing enjoyment over competition may be particularly effective in generating positive exercise experiences. However, individual preference, cultural context, physical capability, and prior experience substantially influence which activities generate positive affect. Therefore, person-centered approaches allowing older adults to explore and select personally meaningful and enjoyable activities may prove most effective. Future research should systematically compare different exercise modalities and program characteristics to identify which features best promote positive exercise experiences and subsequently protect against problematic technology use in older populations.

Second, the early bidirectional relationship underscores the importance of timely prevention. When phone dependence reaches elevated levels, it can initially erode the capacity to enjoy exercise, creating barriers to adopting healthier routines. Early detection of problematic usage combined with simultaneous promotion of exercise enjoyment may therefore be critical in disrupting this cycle before it becomes entrenched.

These findings directly inform public health strategies. Age-friendly exercise initiatives should be co-designed with older adults to ensure activities are enjoyable and foster social bonds, extending beyond purely physical metrics. Digital literacy programs for seniors could be integrated with these wellness activities to create a comprehensive approach. Rather than merely teaching technical skills, such programs could educate participants on managing digital habits, recognizing signs of problematic use, and leveraging technology to support—rather than undermine—an active lifestyle. By integrating these efforts, public health initiatives can harness the protective power of positive exercise experiences to promote healthy digital aging.

### 4.6 Limitations and future directions

Several limitations should be acknowledged. First, the measurement of PEE relied solely on the positive wellbeing subscale of the Subjective Exercise Experience Scale. Although widely used, focusing on a single dimension may narrow conceptual coverage. Future research could employ multifaceted assessments of exercise affect to capture broader experiences. Second, participants were recruited from senior universities, which may not represent the general older population in China. Individuals in such programs may be healthier, more active, and digitally literate than community-dwelling peers. Third, although our design spanned 9 months, a longer follow-up is needed to examine trajectories of behavior and test for delayed reciprocal effects. Fourth, the assessment of smartphone skills relied on self-reported competency verified through brief screening rather than objective skills testing, which may have introduced selection bias by excluding individuals with limited digital literacy who might nonetheless experience problematic phone use patterns. Fifth, although we implemented protocols to minimize coercive pressure, the presence of research assistants during data collection sessions may have created subtle social pressure and influenced responses through social desirability bias. Future studies might consider alternative collection methods, such as sealed envelope procedures or online surveys with delayed submission, to further enhance perceived anonymity. Sixth, while our retention rate was 71.7% over 9 months, participants who dropped out may differ systematically from those who completed all three waves. Our recruitment from senior universities and the provision of small monetary compensation were strategies intended to enhance compliance, but future research in more diverse community settings should employ more robust retention strategies and consider statistical methods to account for attrition bias. Finally, this study did not control for several potential confounders that could influence the relationship between PEE and MPA. For example, physical health status could directly impact an individual’s ability to engage in and enjoy exercise, while also potentially increasing reliance on mobile phones for social connection or distraction if mobility is limited. Similarly, personality traits, such as higher neuroticism or lower extraversion, might predispose individuals to both lower exercise enjoyment and greater tendencies toward addictive behaviors as a coping mechanism. Social support was also not measured, which could independently affect both wellbeing from exercise and the need for digital social surrogates.

It is recommended that subsequent studies measure and control for these variables in order to provide a more nuanced understanding of the observed relationships. Furthermore, such studies may build on these findings by exploring the underlying mechanisms through which PEE protects against MPA, such as enhanced emotion regulation, reduced loneliness, or increased resilience. Multimethod approaches integrating objective activity trackers, smartphone usage logs, and qualitative interviews would allow more nuanced insights. Comparative research across cultures and socioeconomic groups is also needed to verify the robustness of these associations.

## 5 Conclusion

This three-wave longitudinal study provides robust evidence that PEE serves as a stable and significant protective factor against the development of MPA in older adults. While the reverse influence of MPA on PEE was less consistent, our findings highlight the critical role of promoting enjoyable physical activity as a proactive strategy for healthy digital aging. These findings, however, should be viewed in light of their limitations, which also chart a course for future inquiry. These insights extend beyond China, suggesting that global aging policies should integrate enjoyable exercise programs with digital literacy training to foster resilient, technology-balanced lifestyles among older populations.

## Data Availability

The raw data supporting the conclusions of this article will be made available by the authors, without undue reservation.
