# Supplementary material for: The relationship between positive exercise experiences and mobile phone addiction tendencies in older adults: a cross-lagged study
Source: Front Public Health. 2025 Nov 14;13:1710048. doi: 10.3389/fpubh.2025.1710048 (PMC12661996; doi:10.3389/fpubh.2025.1710048)
Supplement: Supplementary file 1 [file Supplementary_file_1.docx]

**Informed Consent Form for Survey Research**

**Study Title:** A Three-Wave Longitudinal Study of Positive Exercise Experience and Mobile Phone Addiction in Older Adults

Institution: School of Physical Education, Jiangxi Normal University
Principal Investigators: Wenying Huang (Corresponding Author), Chang Hu (Corresponding Author)
Contact Information:

- Wenying Huang 18315133143@163.com
- Chang Hu 18046805636@163.com

Ethics Approval: This study has been approved by the Institutional Review Board of the School of Physical Education, Jiangxi Normal University (IRP-JXNU-PEC-2024019), and will adhere to the principles of the Declaration of Helsinki.

1. Background and Purpose

Smartphone use is increasingly common among older adults, raising concerns about problematic use (mobile phone addiction). Positive exercise experiences—feelings of enjoyment, vitality, and well-being during physical activity—may help reduce maladaptive technology use. This three-wave (nine-month) longitudinal survey investigates the dynamic relationship between positive exercise experience and mobile phone addiction among older adults to inform interventions that promote healthy aging.

1. Eligibility and Sample

- Aged 60 years or above; currently enrolled in a senior university; basic smartphone proficiency; able to understand and sign this consent form.
- Participants will be recruited from senior universities in Jiangxi, Hunan, Guizhou, and Chongqing.

1. Procedures and Your Participation

- You will complete questionnaires at three time points (T1: April 2024; T2: September 2024; T3: January 2025). Each session takes approximately 20–30 minutes.
- The questionnaires include demographic items, a Positive Exercise Experience scale, and a Mobile Phone Addiction Tendency scale.
- Data collection will take place in classroom settings with trained staff present to provide assistance. You may complete the survey independently and in a quiet environment.
- Research staff will check completeness; you may ask questions at any time.

1. Time Commitment and Compensation

- Each survey takes about 20–30 minutes.
- You will receive RMB 5 per completed wave as compensation for transportation and time. This token does not constitute undue inducement.

1. Potential Risks or Discomforts

- This is a minimal-risk survey study.
- Some items may cause mild emotional discomfort or fatigue. You may pause, skip any questions you prefer not to answer, or discontinue at any time without penalty.
- Venue safety will be jointly ensured by the host institution and the research team.

1. Potential Benefits

- Direct: Increased awareness of your exercise experiences and phone use habits.
- Indirect: Findings may inform guidance to support healthier technology use and physical activity among older adults.

1. Privacy and Data Security

- Data will be collected anonymously or de-identified. No unnecessary sensitive personal information will be gathered.
- Data are used only for research and teaching; strict confidentiality will be maintained. No disclosure to third parties without your permission unless required by law.
- Paper records will be stored in locked cabinets; electronic data will be stored on encrypted devices. After the legally/ethically required retention period, data will be destroyed per policy.
- Any data shared for academic purposes will be de-identified and aggregated; no personally identifying information will appear in publications or reports.

1. Data Use and Dissemination

- Results may be published in academic journals or presented at conferences. Only aggregate statistics will be reported.
- Upon reasonable request and subject to ethics approval, de-identified data and analysis code may be shared for academic verification.

1. Your Rights

- Voluntary participation: Your decision to participate is entirely voluntary.
- Right to withdraw: You may withdraw at any time for any reason without penalty or loss of benefits.
- Right to be informed: You may ask questions and receive clear answers at any time.
- Privacy rights: You may request deletion of personally identifiable information where legally and ethically permissible.
- Access to results: You may request a summary of overall study findings after the study concludes.

1. Questions and Complaints

- For questions or concerns, contact the investigators (see above).
- For ethics-related concerns, contact the IRB/ ethics committee of the School of Physical Education, Jiangxi Normal University (see official website for contact). Please reference protocol ID IRP-JXNU-PEC-2024019.

1. Consent to Participate
   I have read and understood the information above. The purpose, procedures, potential risks and benefits, privacy protection, and my rights have been explained to me. I have had the opportunity to ask questions and received satisfactory answers. I voluntarily agree to participate in this study and consent to the use of my de-identified data for academic research and publication under the conditions described.

Participant’s Signature: __________________ Date: ____ / ____ / ____
Researcher’s Signature: __________________ Date: ____ / ____ / ____

研究题目：老年人积极运动体验与手机成瘾的交叉滞后研究

研究单位：江西师范大学 体育学院
研究负责人：黄文英（通讯作者），胡畅（通讯作者）
联系方式：

- 黄文英 18315133143@163.com
- 胡昶18046805636@163.com

伦理审批信息：本研究已通过江西师范大学体育学院伦理审查（IRP-JXNU-PEC-2024019），并遵循《赫尔辛基宣言》相关原则。

一、研究背景与目的
随着智能手机在老年人群中的普及，问题性使用（移动电话成瘾）风险受到关注。同时，运动中所体验到的愉悦、活力与积极情绪（积极运动体验）被认为有助于降低不良的技术使用行为。本研究旨在通过连续三次（九个月）跟踪调查，了解老年人的积极运动体验与手机成瘾之间的动态关系，为制定促进健康老龄化的干预策略提供科学依据。

二、参与条件与人数

- 年龄≥60岁；在老年大学就读；具备基本智能手机使用能力；可理解并签署本同意书。
- 本研究计划在江西、湖南、贵州、重庆四省老年大学中招募并随访样本。

三、研究流程与您需要做的事情

- 本研究共进行三次问卷测量（T1：2024年4月；T2：2024年9月；T3：2025年1月），每次约20–30分钟。
- 问卷内容包括：基本信息（如年龄、性别、教育、居住地）、积极运动体验量表、移动电话成瘾倾向量表。
- 测试在老年大学教室进行，现场有研究人员提供说明与必要协助。您可在不受干扰的条件下独立作答。
- 每次测量结束后，由研究人员检查问卷完整性；对于不明白之处，您可随时咨询。

四、时间与补偿

- 每次填写问卷约20–30分钟。
- 每完成一次测量，您将获得5元人民币交通与时间补偿。该补偿为象征性，不构成经济诱导。

五、可能风险与不适

- 本研究为问卷调查，风险极低。
- 个别问题可能引发轻微情绪不适或疲劳。您可随时暂停、跳过不愿回答的问题，或终止参与而无需承担任何责任。
- 场地安全由主办方与研究团队共同保障。

六、潜在受益

- 直接受益：通过答题，您可更了解自身运动感受与手机使用习惯。
- 间接受益：研究结果有助于为老年人提供更合适的运动与数字使用指导，促进身心健康。

七、隐私保护与数据安全

- 本研究采用匿名或去标识化方式记录数据。问卷中不收集无需的敏感个人信息。
- 数据仅用于科研与教学，依法依规严格保密，未经您许可不会向无关第三方披露。
- 纸质问卷保存在加锁柜中，电子数据存储于加密设备，研究结束后在法定或伦理要求的保存期满后按规定销毁。
- 如需学术共享，我们仅共享去标识化汇总数据；任何公开的论文或报告不会披露您的可识别信息。

八、数据使用与结果发表

- 研究结果可能在学术期刊发表或在学术会议报告；内容为汇总统计，不含个人可识别信息。
- 在合理请求与伦理许可下，可能提供去标识化数据与分析代码用于学术复核。

九、您的权利

- 自愿参与：您是否参与完全自愿。
- 随时退出：您可在任何时候、出于任何原因退出研究而不受任何不利影响。
- 知情权：您有权就研究任何问题进行咨询并获得清楚答复。
- 隐私权：您有权要求删除尚可识别的个人信息（在法律与伦理允许的范围内）。
- 获取结果：如您愿意，可向研究团队索取研究的总体结果摘要。

十、疑问与申诉

- 如有任何疑问或不满，您可联系研究负责人（见首页联系方式）。
- 如对研究的伦理问题有疑虑，可联系江西师范大学体育学院伦理委员会（联系渠道以学院官网公示为准），并提供本研究伦理编号IRP-JXNU-PEC-2024019。

十一、同意参加声明
我已阅读并理解以上说明。研究目的、流程、可能风险与受益、隐私保护及我的权利均已获得清楚解释。我有机会就不明之处向研究人员提问并得到满意答复。我自愿参加本研究，并同意在上述条件下使用我的去标识化数据用于学术研究与发表。

**受试者签名：________________ 日期：年__月__日
研究人员签名：____________ 日期：____年__月__日**
